# Fluorescence detection of deep intramucosal cancer excited by green light for photodynamic diagnosis using protoporphyrin IX induced by 5-aminolevulinic acid: an *ex vivo* study

**DOI:** 10.1117/1.JBO.25.6.063809

**Published:** 2020-03-03

**Authors:** Daisuke Ihara, Hisanao Hazama, Takahiro Nishimura, Yoshinori Morita, Kunio Awazu

**Affiliations:** a Osaka University, Graduate School of Engineering, Suita, Japan; b Kobe University Graduate School of Medicine, Department of Gastroenterology, Kobe, Japan; c Kobe University, International Clinical Cancer Research Center, Department of Gastroenterology, Kobe, Japan; d Osaka University, Graduate School of Frontier Biosciences, Suita, Japan; e Osaka University, Global Center for Medical Engineering and Informatics, Suita, Japan

**Keywords:** photodynamic diagnosis, protoporphyrin IX, gastric cancer, intramucosal cancer, fluorescence

## Abstract

**Significance:** The diagnostic depth of photodynamic diagnosis (PDD) for gastric cancer with protoporphyrin IX (PpIX) is limited, which leads to missing intramucosal cancers in screening and surgery.

**Aim:** The reason is that the excitation light, whose wavelength is determined by the highest absorption peak of PpIX (∼405  nm), is strongly attenuated by mucosal tissues. We investigated an excitation wavelength that can extend the diagnostic depth of PpIX fluorescence at the mucosal subsurface.

**Approach:** By calculating the depth-dependent intensity of the excitation light in porcine gastric mucosa for each wavelength, relationships among the wavelength, fluorophore depth, and fluorescence intensity were assessed and fluorescence images of PpIX pellets located at different fluorophore depths were compared experimentally by changing the excitation wavelength.

**Results:** The numerical calculation showed that a 505-nm excitation light provided the highest fluorescence intensities at a fluorophore depth deeper than 1.1 mm. In the fluorescence observation, the fluorescence intensities at fluorophore depths of 0 and 1.0 mm at 405 nm were $5.4\times 10^{3}$ and $1.0\times 10^{3}\;\mathrm{arb.}$ units, whereas those at 505 nm were $5.3\times 10^{1}$ and $1.9\times 10^{2}\;\mathrm{arb.}$ units, respectively.

**Conclusion:** The experimental results suggest that the diagnosis depth of PDD with PpIX for intramucosal cancer can be extended by 505-nm excitation light.

## 1 Introduction

Protoporphyrin IX (PpIX) is biosynthesized from 5-aminolevulinic acid (ALA) to be accumulated selectively in cancer cells. Photodynamic diagnosis (PDD) using PpIX induced by 5-aminolevulinic acid (ALA-PDD), which can visualize and detect cancer, is used for endoscopic diagnosis of various organs. In particular, misdiagnosis in a stomach is dependent on the endoscopist’s skill, and diagnostic accuracy varies among endoscopists.^1^ Therefore, it has been expected that ALA-PDD, which is not dependent on the endoscopist’s skill, can improve the accuracy of gastric cancer detection by reducing the rate of misdiagnosis caused by oversights.^2^

Gastric cancer is classified into two types: differentiated and undifferentiated. Differentiated cancer often occurs from inside of gastric mucosal epithelium. On the other hand, most undifferentiated cancer occurs beneath the epithelium, which is hard to detect since the gastric mucosal epithelial tissue covers the tumor tissues in the endoscopic diagnosis. The accuracy of ALA-PDD might be decreased for the undifferentiated cancer.^3^^–^^5^ In a clinical study, it was reported that 8 of 93 specimens were false-negative in the ALA-PDD and the false-negative specimens consisted of undifferentiated adenocarcinoma.^3^ In the reference, the permeability of the excitation and emission light was discussed as one of the reasons for the false-negative cases. The excitation wavelength of around 405 nm used in current ALA-PDD is at a peak where the PpIX molar extinction coefficient is the largest in the absorption spectrum.^6^^,^^7^ However, the wavelength is strongly affected by the absorption of hemoglobin and the scattering of the mucosa. Thus, compared with the other absorption peak wavelengths of PpIX, the excitation light with the wavelength of around 405 nm is dominated by light attenuation caused by the mucosa. Therefore, it is difficult to detect fluorescence in intramucosal cancer containing undifferentiated cancer because of the strong attenuation of the excitation light. It is important to determine an excitation wavelength by which fluorescence can be detected in ALA-PDD for intramucosal cancer, considering the attenuation of the excitation light by the mucosa.

In a previous study, it was reported that an excitation wavelength of 633 nm was effective for high-glade gliomas in brain by evaluating the effectiveness of the fluorescence detection with dual wavelengths (405 and 633 nm).^8^ The fluorescence was detected using a multichannel spectrometer for the malignant brain tumor. However, fluorescence is generally obtained from an imaging element containing charge-coupled device or complementary metal-oxide-semiconductor (CMOS) in ALA-PDD. Using the imaging element, the fluorescence in the wavelength range that overlaps the excitation wavelength is difficult to detect because the excitation wavelength range should be cut off for fluorescence detection. To filter out the excitation light, the emitted fluorescence cannot be detected in the wavelengths where the spectra of the excitation light and the fluorescence emission are overlapped. It is estimated that an excitation light that overlaps the fluorescence wavelength around 633 nm cannot be used in ALA-PDD. In this study, the purpose was to expand the diagnostic depth of PpIX fluorescence by determining an excitation wavelength whose light can excite PpIX effectively. Therefore, the relationships among the depth of PpIX, wavelength, and fluorescence intensity were assessed based on attenuation of the excitation light by the mucosa and the absorption characteristics of PpIX, and the excitation wavelength by which the fluorescence intensity of PpIX was maximized at each fluorophore depth was investigated. The effectiveness of the excitation wavelength, which maximized fluorescence intensity at the gastric subsurface, was evaluated by an *ex vivo* experiment using porcine gastric mucosa compared with the wavelength of 405 nm in the current ALA-PDD.

## 2 Materials and Methods

### 2.1 Determination of an Excitation Wavelength that Maximizes the Fluorescence Intensity Based on Tissue Optics

#### 2.1.1 Theory

When $\mu_{a}\ll 3\mu_{s}^{\prime }$, light penetration depth δ in biological tissue is $\delta =1/\sqrt{3\mu_{a}\{\mu_{a}+\mu_{s}^{\prime }\}}$, where $\mu_{a}$ is the absorption coefficient and $\mu_{s}^{\prime }$ is the reduced scattering coefficient.^9^ The reduced scattering coefficient $\mu_{s}^{\prime }$ is calculated by $\mu_{s}^{\prime }=\mu_{s}(1-g)$ using the scattering coefficient $\mu_{s}$ and anisotropy factor g. Therefore, when it is assumed that uniform light is irradiated on a mucosal surface, the light intensity $I_{z}$ at depth z from the mucosal surface to PpIX (fluorophore depth) is given as

$$I(\lambda ,z)=I_{0}\;\exp[-\frac{z}{\delta (\lambda )}],$$ (1)

where δ(λ) is the light penetration depth as a function of the wavelength λ, and $I_{0}$ is the light intensity after entering the mucosa. When the concentration of PpIX is low and the beam path is short, it can be considered that attenuation of the excitation light in PpIX is negligible. Thus, at a low excitation light intensity, the fluorescence intensity of PpIX is approximately proportional to the excitation light intensity. The fluorescence intensity F(λ) is expressed as

F(λ)=AΦε(λ)CLI, (2)

where I is the excitation light intensity, Φ is the quantum yield of the fluorescence, ε(λ) is the molar extinction coefficient of PpIX, L is the length of the PpIX volume, C is the concentration of PpIX, and A is the fraction of the available light collected.^10^ Hence, the fluorescence intensity F(λ,z) with each wavelength at fluorophore depth z is determined by Eqs. (1) and (2):

$$F(\lambda ,z)=A\Phi \varepsilon (\lambda )CLI_{0}\;\exp[-\frac{z}{\delta_{\mathrm{ex}}(\lambda )}],$$ (3)

where $\delta_{\mathrm{ex}}(\lambda )$ is the excitation light penetration depth.

In the visible range, the light penetration depth of the fluorescence of PpIX is not dependent on the excitation wavelength because the fluorescence spectrum of PpIX has the same shape at various excitation wavelengths. Therefore, F(λ,z) is attenuated by the mucosa and is reduced to fluorescence intensity $F_{\mathrm{suf}}(\lambda ,z)$ obtained on the mucosal surface. The fluorescence intensity $F_{\mathrm{suf}}(\lambda ,z)$ takes the form

$$F_{\mathrm{suf}}(\lambda ,z)=F(\lambda ,z)\exp[-\frac{z}{\delta_{f}(\lambda )}],$$ (4)

where $\delta_{f}(\lambda )$ is the fluorescence penetration depth. In this study, $\delta_{f}(\lambda )$ was approximated as a monochromatic wavelength of 644 nm, which is the fluorescence center wavelength of the PpIX. Therefore, based on Eqs. (3) and (4), the fluorescence intensity $F_{\mathrm{suf}}(\lambda ,z)$ obtained on the mucosal surface can be modeled using the equation:

$$F_{\mathrm{suf}}(\lambda ,z)=A\Phi \varepsilon (\lambda )CLI_{0}\;\exp[-\frac{z}{\delta_{\mathrm{ex}}(\lambda )}]\exp[-\frac{z}{\delta_{f}(\lambda )}].$$ (5)

Thus, factors (A, Φ, C, L, and $I_{0}$) that are constant can be canceled when using the ratio of the fluorescence intensity.^11^ The ratio of the fluorescence intensity Γ(λ,z) is defined as

$$\Gamma (\lambda ,z)=\frac{F_{\mathrm{suf}}(\lambda ,z)}{F_{\max}},$$ (6)

where $F_{\max}$ is the maximum fluorescence intensity on the mucosal surface, i.e., the maximum value of $F_{\mathrm{suf}}(\lambda ,0)$. By using Γ(λ,z), evaluation of the extension possibility of the detection depth is not be affected by the difference of the PpIX accumulation amount in cancer cells and the imaging system performance.

The fluorescence bandwidth of PpIX has a wavelength range of 600 to 740 nm.^2^ Thus, we investigated the excitation wavelength by which the fluorescence intensity was maximized for application to ALA-PDD in the visible range (wavelength range of 400 to 600 nm) that did not overlap with the fluorescence bandwidth of PpIX. The ratio of the fluorescence intensity Γ(λ,z) (wavelength resolution: 1 nm; fluorophore depth resolution: 0.1 mm) was calculated by Eq. (6) when irradiating the excitation light to PpIX beneath the mucosal surface. The excitation wavelength by which the fluorescence intensity was maximized was derived as the wavelength by which Γ(λ,z) was the largest in the wavelength range of 400 to 600 nm at each fluorophore depth.

#### 2.1.2 Measurement of the molar extinction coefficient of the PpIX solution

The molar extinction coefficient of a PpIX solution was measured for numerical calculation. PpIX powder (P8293-1G, Sigma-Aldrich, St. Louis, Missouri) was dissolved in dimethyl sulfoxide (D4540-100ML, Sigma-Aldrich) at a concentration of 10  μM. The PpIX solution (3 mL) was added to a quartz cell (F15-UV-10, As One, Japan) with an optical path length of 10 mm. The molar extinction coefficient ε(λ) of the PpIX solution was then measured by a spectrophotometer (U3500, Hitachi, Japan).

#### 2.1.3 Tissue preparation

Porcine stomach was purchased from IVTeC Co., Ltd. (Tokyo, Japan) and it was derived in refrigerated storage within 3 days after the animal was sacrificed. Using a shape-edged knife, each sample was trimmed into an approximate 2×2  cm square. The samples were immediately rinsed briefly in saline to remove mucus. Prepared samples were wrapped in aluminum foil, sealed, and frozen at −25°C until they were used.

#### 2.1.4 Measurement of the absorption and scattering coefficients of porcine gastric mucosa

The absorption and scattering coefficients of porcine gastric mucosa were measured for numerical calculation. Trimmed porcine gastric wall was used. Only porcine gastric mucosa was separated from the porcine gastric wall by a cryotome (CM1850, Leica, Germany). The thickness of the mucosa was adjusted using glass slides with a thickness of 1 mm and spacers with a thickness of 0.5 mm. Diffuse reflectance and total transmittance of the mucosa were measured at an integrating time of 400 ms with a double integrating sphere optical system. The absorption and scattering coefficients of the mucosa were calculated from the diffuse reflectance and total transmittance using the inverse Monte Carlo method.^9^

### 2.2 *Ex Vivo* Evaluation of the Excitation Wavelength Effectiveness

#### 2.2.1 Measurement setup

**Fig. 1:**
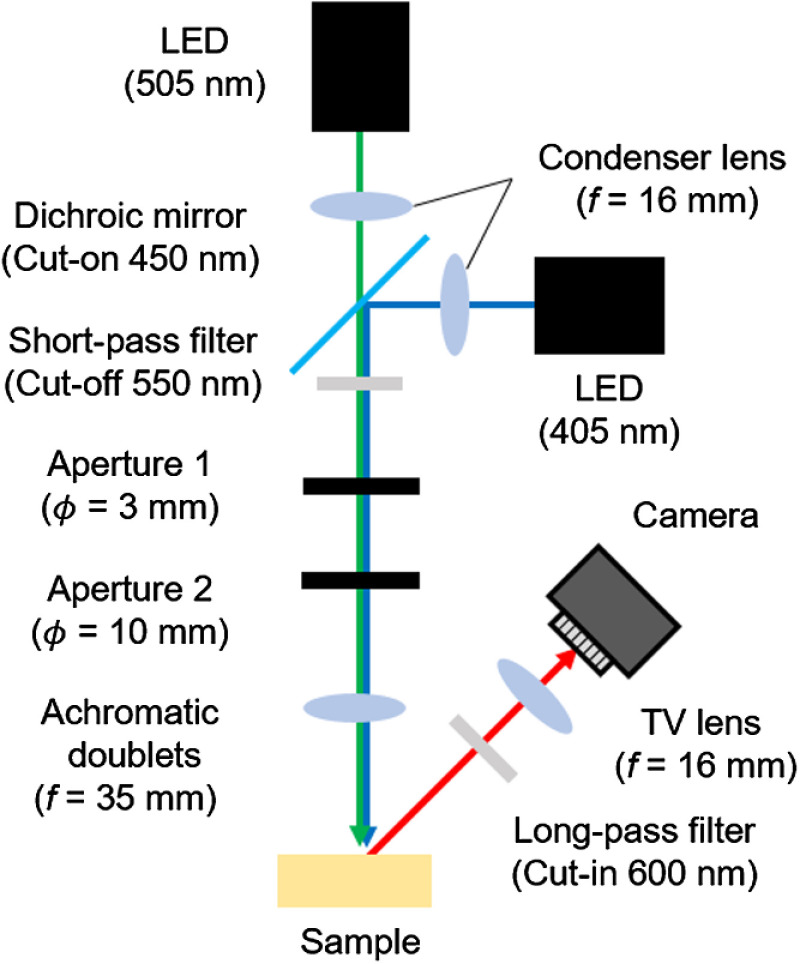
Setup for the fluorescence observation. f is the focal length, and ϕ is the aperture diameter. The excitation lights of dual wavelengths of 405 and 505 nm can be switched. Uniform light is irradiated to a sample surface with this optical system based on Kohler illumination. The long-pass filter is set in front of the TV lens to obtain only fluorescence of PpIX.

The experimental setup for fluorescence observation is shown in Fig. 1. A blue-violet LED (M405L3, Thorlabs, Newton, New Jersey) with a nominal wavelength of 405 nm and green LED (M505L3, Thorlabs) with a nominal wavelength of 505 nm were used as light sources. The excitation wavelength used in current ALA-PDD and the excitation wavelength by which the fluorescence was maximized at the subsurface calculated by equations in Sec. 2.1.1 were irradiated to the sample. To irradiate uniform excitation lights with a beam width of 4 mm defined as the D86 width to the sample surface, a fluorescence observation system was designed based on Kohler illumination with a condenser lens (ACL25416U-A, Thorlabs), achromatic doublets (AC254-0350A, Thorlabs), and two apertures.^12^ The beam paths of dual wavelengths of 405 and 505 nm were combined by a dichroic mirror (69-898, Edmund Optics, Barrington, New Jersey) in front of the light sources. A short-pass filter was set to cut off excitation light after wavelengths of 550 nm. Therefore, the excitation wavelength irradiated to the sample did not overlap with the fluorescence wavelength of PpIX. In the experiment, a CMOS camera (83-770, Edmund Optics) and TV lens (C1614-A, Pentax, Japan) were used to observe the sample. The F value of the TV lens was set to 2.8. In the fluorescence observation, a long-pass filter (FELH600, Thorlabs) was set in front of the TV lens to obtain only fluorescence.

#### 2.2.2 Sample preparation

A PpIX pellet consisting of PpIX solution and clear epoxy resin (87136, Tamiya, Japan) was used as the fluorophore. PpIX powder was dissolved in dimethyl sulfoxide at a concentration of 1 mM. The PpIX solution was mixed with the clear epoxy resin. The final PpIX concentration was set as 10  μM according to the previous studies reporting that PpIX concentration was 10 to 20  μM in gastric tumors of rat.^13^^,^^14^ The mixed solution was heated in a water bath at 80°C within about 1 min for deaeration. A PpIX sheet with a thickness of 1 mm was prepared using the solution, two glass slides, and spacers with a thickness of 1 mm. After hardening the PpIX sheet for about 48 h, the PpIX sheet was removed from the glass slides. Then, a clear PpIX pellet with a concentration of 10  μM, a thickness of 1 mm, and a diameter of 2.5 mm was prepared to hollow out the PpIX sheet by a hole punch. The absorption coefficient of the PpIX pellet was measured as shown in Sec. 2.1.2.

**Fig. 2:**
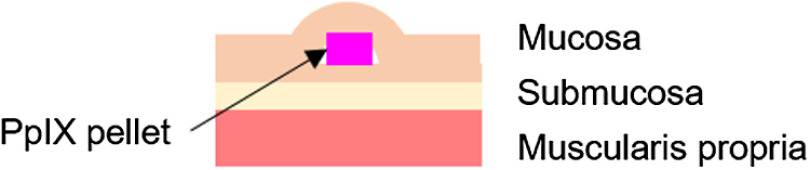
Schematic diagram of the sample structure. The diameter of the PpIX pellet was 2.5 mm, its thickness was 1 mm, and the PpIX concentration was 10  μM.

A schematic diagram of the prepared sample structure is shown in Fig. 2. Porcine gastric wall was prepared as indicated in Sec. 2.1.3. The PpIX pellet was set on the mucosal surface for fluorescence observation on the mucosal surface (fluorophore depth of 0 mm). After freezing and fixing the sample with a specimen matrix (Tissue-Tek OCT compound, Sakura Finetek Japan, Japan), the porcine gastric mucosa was sectioned at an arbitrary depth with a cryotome. The PpIX pellet was set between the upper and lower sections of the mucosa as shown in Fig. 2 for fluorescence observation of the PpIX pellet at the mucosal subsurface.

#### 2.2.3 Fluorescence observation

The power density of excitation lights with dual wavelengths of 405 and 505 nm was set to $140\;\mathrm{mW}/{\mathrm{cm}}^{2}$ on the mucosal surface using a power sensor that combined a sensor head (30A-BB-18, Ophir Optronics, Israel) and display (Nova II, Ophir Optronics). Excitation lights of each wavelength were irradiated to the sample surface at the same power density. Fluorescence of the PpIX pellet set on the mucosal surface or at the subsurface was observed by the fluorescence observation system described in Sec. 2.2.1. To observe fluorescence with an intensity range over several orders, the exposure time was set between 2.0 and 46.4 ms. The fluorescence observations were performed once at each fluorescence depth since the sample preparation to make the sample has the same depth was difficult due to the use of manually sliced sample. Background values were subtracted from the obtained fluorescence images. The background values were obtained from the sample without the pellet. For comparison, fluorescence intensities were obtained by calibrating the obtained fluorescence images using a linearity of the detected signal to exposure time. The calibration curve was obtained, and the linearity was confirmed in advance. After the fluorescence observation, images were obtained under white light by the camera without the long-pass filter to observe the mucosal shape. No fluorescence was measured when the pellet containing no PpIX was excited by the 405- or 505-nm wavelength light.

After freezing and fixing the sample with the specimen matrix, the sample was sectioned to a thickness of 50  μm by the cryotome. The position of the PpIX pellet beneath the surface was identified to analyze the section of the sample by hematoxylin and eosin staining, and the depth of the PpIX position from mucosal surface to the pellet was measured by a slide scanner (NanoZoomer 2.0 RS, Hamamatsu Photonics, Japan). The depth of the PpIX position was obtained by averaging the measured depths at different 15 points for each sample. The attenuation rate of fluorescence was evaluated with respect to each fluorophore depth by the calibrated fluorescence intensity.

## 3 Results

### 3.1 Determination of the Excitation Wavelength that Maximizes Fluorescence Intensity Based on Tissue Optics

#### 3.1.1 Measurement value of parameters using numerical calculation

**Fig. 3:**
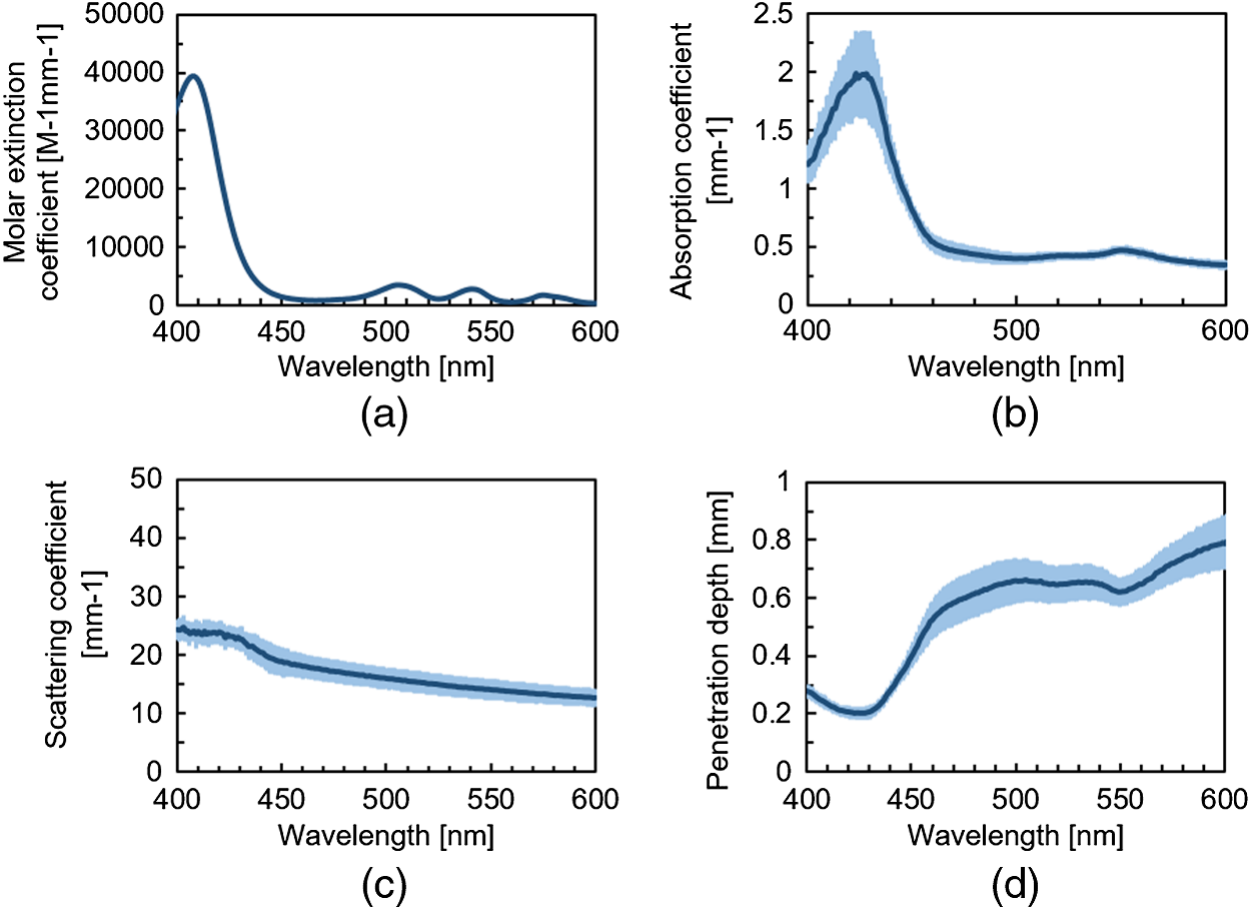
(a) PpIX absorption spectrum (n=3). (b) Absorption and (c) scattering spectra of porcine gastric mucosa (n=3). (d) Excitation light penetration depth in porcine gastric mucosa (n=3). Each error bar in (b)–(d) indicates the standard deviation obtained from gastric mucosa at three different locations.

Figure 3(a) shows the measured spectrum of the molar extinction coefficient of the PpIX solution. Absorption peaks of PpIX solutions were observed at wavelengths of 408, 505, 541, 575, and 630 nm. Figures 3(b) and 3(c) show the absorption and scattering spectra of the porcine gastric mucosa, respectively. In Fig. 3(b), absorption peaks were detected around wavelengths of 420 and 550 nm because of the absorption of hemoglobin.^15^ In Fig. 3(c), compared with the long wavelength side, the scattering coefficient was highly observed on the short wavelength side. Figure 3(d) shows excitation light penetration depths in porcine gastric mucosa calculated from the absorption and scattering spectra in Figs. 3(b) and 3(c). The excitation light penetration depth at the wavelength of 405 nm used in the current ALA-PDD was 0.26 mm, which was more difficult to penetrate the mucosa than the other PpIX absorption peaks.

The absorption coefficient $\mu_{a}$, and reduced scattering coefficient $\mu_{s}^{\prime }$ at a wavelength of 635 nm were 0.26 and $1.2\;{\mathrm{mm}}^{-1}$, respectively. The fluorescence penetration depth $\delta_{f}(\lambda )$ was calculated as 0.98 mm from the optical properties.

#### 3.1.2 Derivation of the excitation wavelength by which fluorescence intensity is maximized at each fluorophore depth using measured parameters

**Fig. 4:**
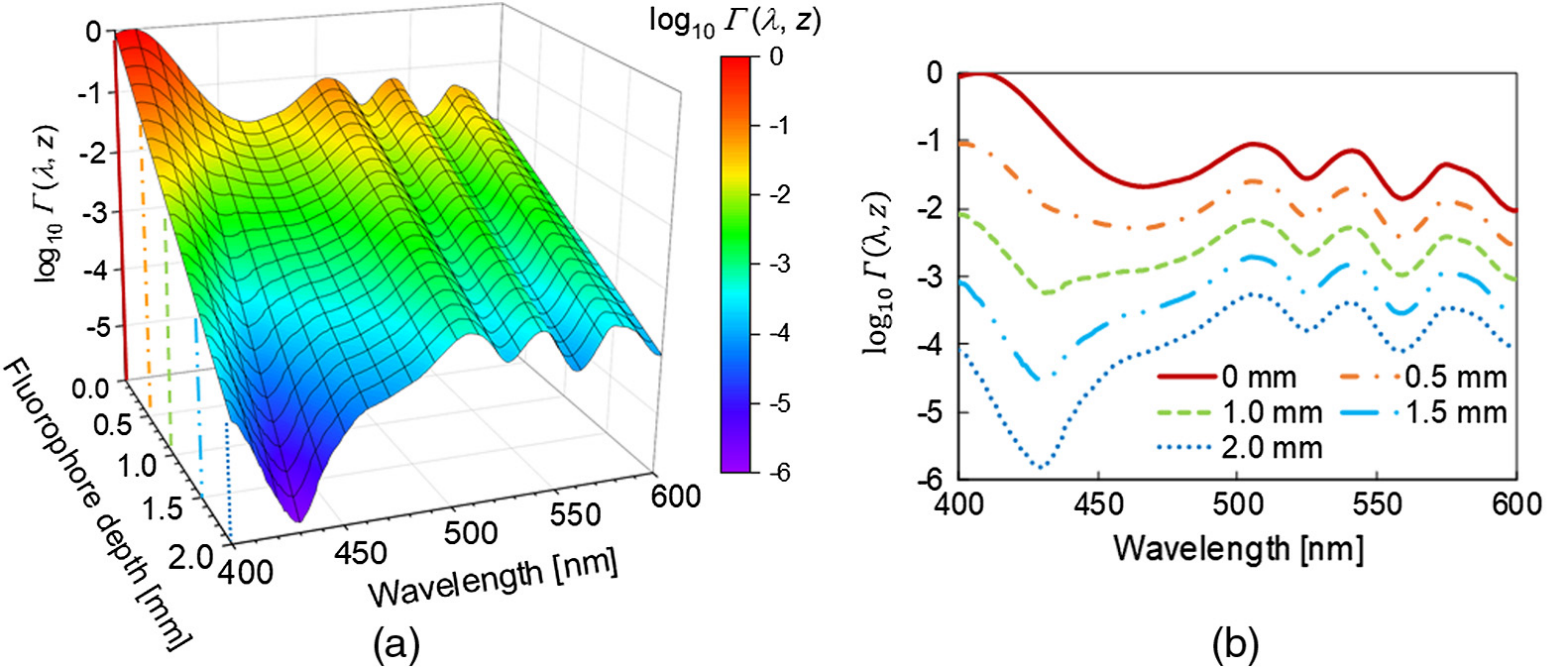
(a) Relationship between the fluorescence intensity and fluorophore depth in the wavelength range of 400 to 600 nm. (b) Cross section of (a) at fluorophore depths of 0, 0.5, 1.0, 1.5, and 2.0 mm. At a fluorophore depth shallower than 1.1 mm, the most effective wavelength was within the blue-violet light range. At a fluorophore depth deeper than 1.1 mm, the most effective wavelength was 505 nm.

The relationships among the excitation wavelength, fluorophore depth, and the ratio of fluorescence intensity Γ(λ,z) are shown in Fig. 4. The ratio of the fluorescence intensity Γ(λ,z) at the wavelength of 408 nm was the highest in the wavelength range of 400 to 600 nm at the mucosal surface (z=0). At a fluorophore depth shallower than 1.1 mm, the wavelengths with the highest fluorescence intensity shifted to shorter wavelengths as the fluorophore depth increased. However, at a fluorophore depth deeper than 1.1 mm, the wavelength with the highest fluorescence intensity was 505 nm.

The excitation light penetration depth at the wavelength of 405 nm used in the current ALA-PDD was about three times shallower than that at the wavelength of 505 nm. Moreover, the attenuation rate in the mucosa at the wavelength of 405 nm was about 20 times higher than that at the wavelength of 505 nm. Hence, by comparing wavelengths of 405 and 505 nm, the fluorescence intensity with the wavelength of 405 nm was higher than that with the wavelength of 505 nm at a fluorophore depth shallower than 1.0 mm. However, the fluorescence intensity with the wavelength of 505 nm was higher than that with the wavelength of 405 nm at a fluorophore depth deeper than 1.0 mm. Based on the above result, using the excitation light with the wavelength of 505 nm was more effective than with wavelength of 405 nm in ALA-PDD for intramucosal gastric cancer.

### 3.2 *Ex Vivo* Evaluation of the Excitation Wavelength Effectiveness

**Fig. 5:**
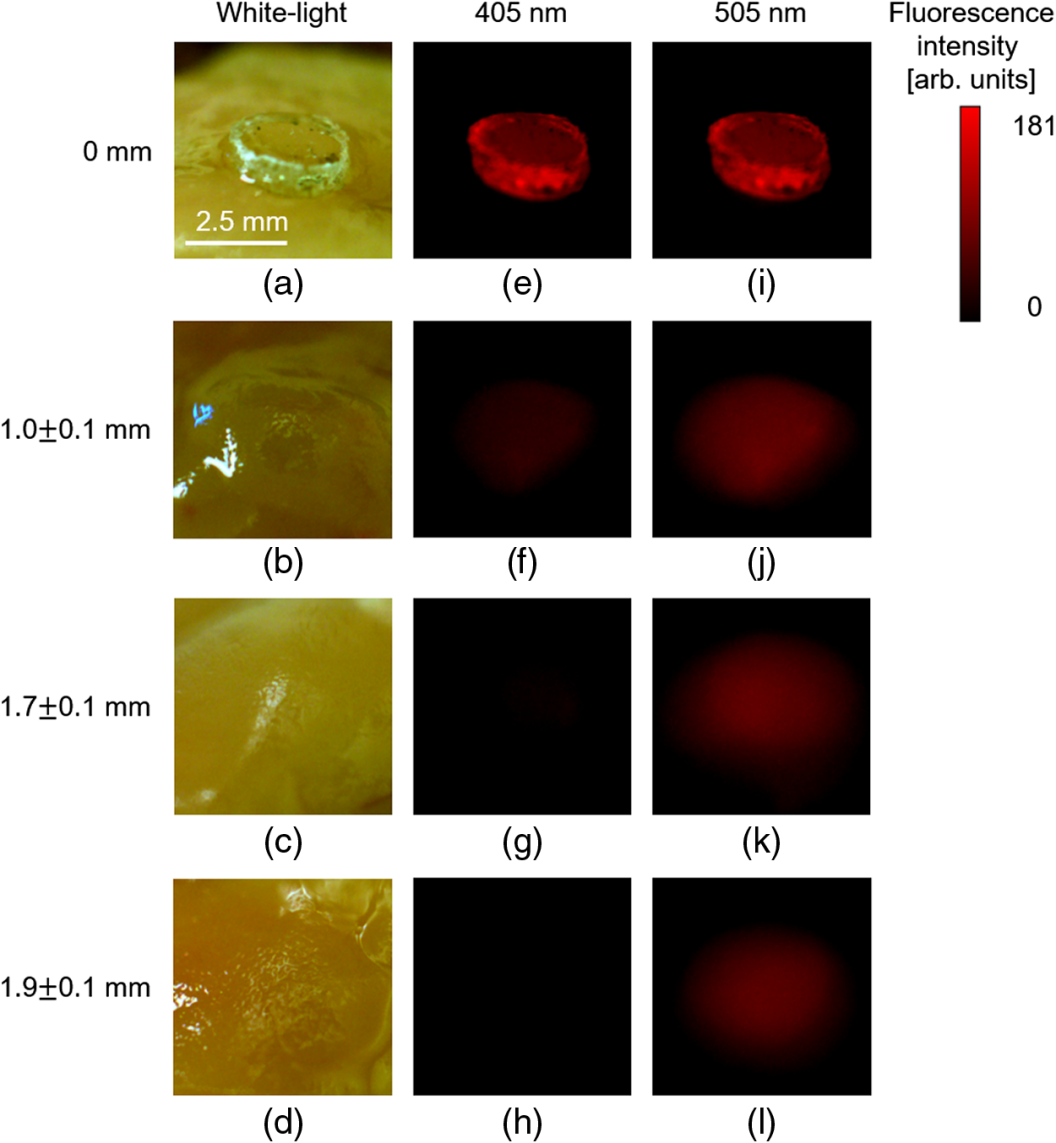
Obtained images of samples set at fluorophore depths of (a), (e), and (i) 0 mm; (b), (f), and (j) 1.0±0.1  mm; (c), (g), and (k) 1.7±0.1; and (d), (h), and (l) 1.9±0.1  mm. (a)–(d) White light images and individual fluorescence images with each excitation wavelengths of (e)–(h) 405 nm and (i)–(l) 505 nm. The exposure time was set at (e) 2 ms; (i) 10 ms; (f) 40 ms; (j) 25 ms; and (g), (k), (h), and (l) 46.4 ms. Background values were subtracted for each fluorescence image. No fluorescence was observed when the pellet containing no PpIX was excited by the 405- or 505-nm wavelength light (data not shown).

Figure 5 shows white light and fluorescence images obtained. Figure 5(e) is a fluorescence image when the excitation light with the wavelength of 405 nm was irradiated to the PpIX pellet on the mucosal surface. In Fig. 5(e), the calibrated fluorescence intensity was $5.4\times 10^{3}\;\mathrm{arb.}$ units. Figure 5(i) is a fluorescence image when the excitation light with the wavelength of 505 nm was irradiated to the PpIX pellet on the mucosal surface. In Fig. 5(i), the calibrated fluorescence intensity was $1.0\times 10^{3}\;\mathrm{arb.}$ units. The fluorescence intensity with the wavelength of 405 nm was about six times higher than that with the wavelength of 505 nm. Therefore, the numerical calculation and *ex vivo* experiment revealed that the excitation light with the wavelength of 405 nm used in the current ALA-PDD was effective for PpIX on the mucosal surface. Figures 5(f) and 5(j) are fluorescence images when excitation lights with wavelengths of 405 and 505 nm were irradiated to the PpIX pellet at the subsurface with a fluorophore depth of 1.0±0.1  mm. In Figs. 5(f) and 5(j), the calibrated fluorescence intensities were 5.3×10 and $1.9\times 10^{2}\;\mathrm{arb.}$ units, respectively. In the experiment, the fluorescence intensity at the excitation wavelength of 505 nm became higher than that excited at 405 nm as the pellet position got deeper due to the mucosal effect. Figures 5(g) and 5(k) are fluorescence images when excitation lights with wavelengths of 405 and 505 nm were irradiated to the PpIX pellet at the subsurface with the fluorophore depth of 1.7±0.1  mm. In Figs. 5(g) and 5(k), the calibrated fluorescence intensities were 1.5×10 and 7.5×10  arb. units, respectively. Figures 5(h) and 5(l) are fluorescence images when excitation lights with wavelengths of 405 and 505 nm were irradiated to the PpIX pellet at the subsurface with the fluorophore depth of 1.9±0.1  mm. In Figs. 5(h) and 5(l), the calibrated fluorescence intensities were 0 and 6.8×10  arb. units, respectively. No fluorescence with the wavelength of 405 nm was detected in Fig. 5(h). Based on the above result, it was clarified that the absorption and scattering in tissue at the wavelength of 405 nm had a strong influence on attenuation of the fluorescence intensity. Furthermore, the fluorescence intensity beneath the mucosal surface with the excitation wavelength of 505 nm became higher than that with the excitation wavelength of 405 nm in both the calculation and experiment.

**Fig. 6:**
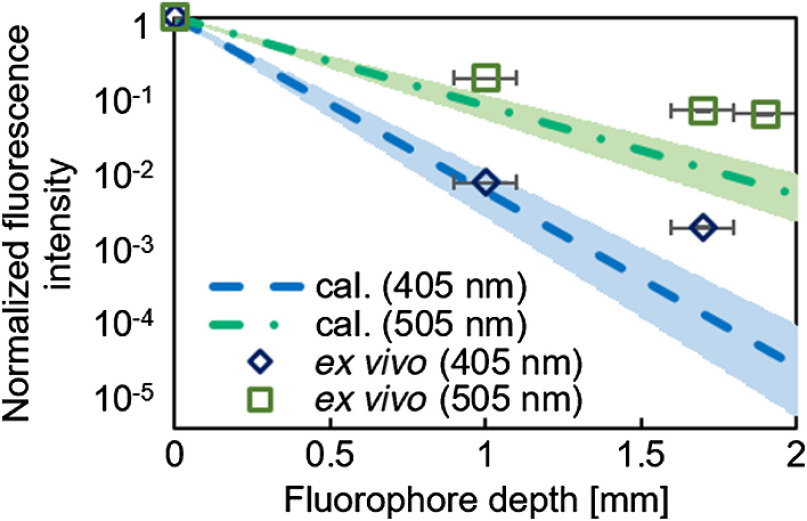
Normalized fluorescence intensity against the fluorophore depth. *Ex vivo* normalized fluorescence intensity was obtained by a single measurement. The fluorescence intensity with the excitation wavelength of 405 nm at a fluorophore depth of 1.9 mm was not included because it was 0. Error bars in x axis indicate the standard deviations of the fluorophore depth. Blue and green colored areas indicate the ranges between the maximum and minimum normalized fluorescence intensity calculated using the pairs of the maximum absorption and scattering coefficients and the minimum absorption and scattering coefficients of the standard deviations shown in Fig. 3.

Figure 6 shows the relationship between the normalized fluorescence intensity of each excitation wavelength (405 and 505 nm) and the fluorophore depth in the numerical calculation and *ex vivo* experiment. Fluorescence attenuation in the mucosa with the wavelength of 505 nm was lower than that with the wavelength of 405 nm in both the numerical calculation and *ex vivo* experiment. Hence, using the excitation wavelength of 505 nm may improve the detection depth in ALA-PDD for intramucosal cancer.

## 4 Discussion

The fluorescence intensity beneath mucosal surface with the excitation wavelength of 505 nm became the highest in the wavelength range of 400 to 600 nm from the numerical calculation based on the optical properties of gastric mucosa and the *ex vivo* experiment. PpIX distributed in realistic cancer is not uniform actually unlike the pellet used in the *ex vivo* experiment. The similar result which the 505-nm excitation light is effective, however, will be found in tissue with heterogeneous distribution of PpIX since the heterogeneous distribution is canceled in comparing the attenuation rates at each fluorophore depth.

The fluorescence intensities at mucosal surface (fluorophore depth of 0 mm) were compared between the 405- and 505-nm excitation lights by evaluating the obtained fluorescence images before normalized. The fluoresce intensity with the wavelength of 405 nm was observed as about six times higher than that with the wavelength of 505 nm in the *ex vivo* experiment. On the other hand, in the numerical analysis, the fluorescence intensity by the 405-nm light excitation was calculated as about 11 times higher than that by the 505-nm light excitation. The difference between the *ex vivo* experimental result and the numerical calculation is explained by the following reasons. One is the difference of the two LED bandwidths. The full-width at half-maximum (FWHM) of the 405-nm LED was ±20  nm. However, the FWHM of the 505-nm LED was ±30  nm, which excites the PpIX absorption peak not only at around 505 nm but also at near another peak around 541 nm [Fig. 3(a)]. Therefore, the experimentally measured fluorescence intensity with the 505-nm LED was larger than the calculated values. The second considerable reason is the attenuation of the excitation light in the pellet. In general, when stronger fluorescence is emitted as the absorption coefficient increases, the excitation light is absorbed by PpIX at a large volume to be attenuated.^10^ Although the attenuation of the excitation light by PpIX may have occurred in the *ex vivo* experiments, in the numerical calculation, the attenuation of the excitation light by PpIX was ignored in the calculation. The absorption coefficients of PpIX in the pellet were $2.59\times 10^{-1}\;{\mathrm{mm}}^{-1}$ at a wavelength of 405 nm and $2.33\times 10^{-2}\;{\mathrm{mm}}^{-1}$ at a wavelength of 505 nm. The absorption coefficients of the epoxy resin were $1.93\times 10^{-2}\;{\mathrm{mm}}^{-1}$ at a wavelength of 405 nm and $9.26\times 10^{-3}\;{\mathrm{mm}}^{-1}$ at a wavelength of 505 nm. From these values, the penetration depth of the wavelength of 405 nm was shorter than 505 nm in the PpIX pellet. The difference of the light penetration depth between two wavelengths affected the excitation of PpIX in the pellet.

In Fig. 6, the attenuation rates in the *ex vivo* experiment were reduced compared with the numerical calculation values. There are the following possible reasons. The first possible reason is the measurement error of the optical properties. Previous studies reported that the absorption coefficient of soft tissues and vessels *in vitro* is larger than the actual one because hemoglobin concentration per volume increases by compression and dehydration sample in preparation.^16^^,^^17^ The sliced mucosal section measured by the double integrating sphere optical system was slightly compressed by glass slides to adjust the section with the glass slides. The sample was also more sensitive to dehydration because the thickness of the measured sample (0.5 mm) was thinner than the gastric wall used in the *ex vivo* experiment. Therefore, the measured absorption coefficient could be higher than the one used in the *ex vivo* experiment, which caused the difference between numerical calculation and *ex vivo* experiment. The second reason is Fresnel reflection. The Fresnel reflection was not considered because PpIX was assumed to be in tumor tissue and $I_{0}$ was the light intensity after entering the mucosa in the numerical calculation. However, in the *ex vivo* experiment, the Fresnel reflection may have occurred because of the difference between the refractive indexes of the pellet and mucosa. In general, the refractive index of epoxy resin is ∼1.48, which is higher than that of biological tissue.^18^ In the case of the PpIX pellet on the mucosal surface, ∼19.3% of the excitation light and fluorescence were lost because of the Fresnel reflection of irradiation light and fluorescence at the interface (air–PpIX pellet). The incident angle, refractive index of tissue, and refractive index of air were assumed to be 0 deg, 1.33, and 1.00, respectively. However, in the case of the PpIX pellet at the mucosal subsurface, ∼18.8% of the excitation light and fluorescence were lost because of the Fresnel reflection of irradiation light and fluorescence at the interface (air–mucosa) and (mucosa–PpIX pellet). Therefore, the fluorescence was more strongly attenuated by the Fresnel reflection in the case of the PpIX pellet on the mucosal surface than in the case of the PpIX pellet at the mucosal subsurface. Based on the above discussion, it was assumed that the attenuation rates of the fluorescence intensity in the *ex vivo* experiment were lower than those in the numerical calculation. Another reason is the calibration error, which occurred when the dynamic range was extended using the linear response of the used camera with light intensity. The possible maximum calibration error was estimated as 0.8%.

Roberts et al. reported that the malignant brain tumor at the subsurface (up to the fluorophore depth of 5 mm from the surface) can be detected by fluorescence of PpIX with red-light excitation (the wavelength of 630 nm).^19^ The attenuation rates in using red-light excitation were calculated for intramucosal gastric cancer as well as the case of the 405- and 505-nm wavelength light excitation. The attenuation rate with the wavelength of 630 nm was higher at the fluorophore depth of 1.2 mm or deeper region compared with the wavelength of 405 nm. At the fluorophore depth of 2.3 mm or deeper, the attenuation rate with the wavelength of 630 nm was higher than that with the wavelength of 505 nm. In addition, when using the excitation wavelength around 630 nm in PDD, the fluorescence is reduced by about 50% because some fluorescence wavelength is cut off by the band-pass or long-pass filter. From the above, in the tumor detection in gastric mucosal subsurface, the wavelength of 505 nm may have the advantage compared with the red light because the thickness of the gastric mucosa is 1.4±0.3  mm, which is thinner than the thickness of the brain.^20^

Salomatina and Yaroslavsky^21^ reported that absorption coefficient at a wavelength of 405 nm was ∼30% less *ex vivo* than *in vivo* while that at a wavelength of 505 nm was ∼15% *ex vivo* than *in vivo* because of the loss of blood. It means that the actual absorption coefficients at 405 and 505 nm *in vivo* are larger than that used in the numerical calculation due to the increase of the amount of hemoglobin. Increase of the amount of hemoglobin *in vivo* mainly affects the attenuation of the fluorescence intensity at the wavelength of 405 nm compared to 505 nm. It implies that the wavelength of 505 nm is effective compared to 405 nm *in vivo* at the shallower fluorophore depth than 1.0 mm, which was obtained in the numerical calculation. In this study, the optical properties of mucosal lesions were not simulated in the pellet due to the following reasons. First, the optical properties of mucosal lesions were difficult to measure. Second, the optical properties of the pellet are difficult to be adjusted at the two excitation wavelengths and fluorescence wavelengths at the same time. Therefore, the experiments *in vivo* will be required to evaluate the effect of the optical properties in the lesions in future clinical studies. In the numerical simulation, PpIX was assumed to be distributed homogeneously in lesion tissues. The optical properties used in the simulations are the values that contain the effect of blood. As shown in Fig. 6, the fluorescence intensities by the numerical simulation were lower than that by the *ex vivo* experiment. One of the reasons is that there was no effect of blood in the pellet. If blood is added to the pellet, the 405-nm excitation light is more attenuated compared to the 505-nm excitation light due to the strong absorption of hemoglobin. Therefore, it is inferred that 505-nm light excitation is more effective than 405-nm light excitation because the excitation light with 405 nm is further attenuated. ^15^^,^^21^

The PpIX fluorescence background from normal tissue may affect the detection contrast in clinical use. PpIX concentration ratio between tumor and normal tissues (T/N ratio) has been reported as about 11.^22^ So far, a clinical study reported ALA-PDD for brain tumor by red light excitation can be achieved to collect PpIX fluorescence associated with subsurface tumor at the depth up to around 5 mm.^19^ On the other hand, thickness of gastric mucosa is 1.4±0.3  mm and the volume of normal tissue related to the fluorescence background in the mucosa is smaller than the case for detecting subsurface tumor. Each excitation wavelengths have the same fluorescence ratio between tumor and normal tissues since because the fluorescence intensity is proportional to the concentration. The PpIX fluorescence background for gastric cancer observation will provide less damage of the contrast than the clinical case.^19^ Thus, although the PpIX fluorescence background damages the contrast of tumor detection, green light excitation will distinguish tumor lesion in normal gastric mucosa.

Our results are useful for humans because the thickness of human gastric mucosa is 1.4±0.3  mm.^20^ The light penetration depth in the human gastric mucosa is almost identical to the light penetration depth in the porcine gastric mucosa measured in this study, and the light in the blue-violet range is less likely to penetrate the human mucosa than the porcine mucosa compared with light with the wavelength of 505 nm.^23^ Therefore, the fluorescence intensity beneath mucosal surface with the excitation wavelength of 505 nm may become the highest in the wavelength range of 400 to 600 nm at a shallower fluorophore depth in the human stomach than in the porcine stomach. From the above discussion, the excitation light with the wavelength of 505 nm is effective in ALA-PDD for intramucosal cancer such as scattered undifferentiated adenocarcinoma, which could not be detected by the current ALA-PDD with the wavelength of 405 nm.^3^^–^^5^ In addition, using the excitation wavelength of 505 nm in the ALA-PDD may be useful to for gastric cancer covered over non-neoplastic epithelium after *Helicobacter pylori* eradication therapy, which is difficult to diagnose by the current endoscopy.^24^^–^^27^

The absorption and scattering coefficients of other mucosal tissues, such as cervical tissue, colon tissue, and maxillary mucous membrane, have spectrum shapes similar to those of the gastric mucosa.^28^^–^^30^ The absorption peaks of the gastric mucosa are wavelengths of around 420 and 550 nm, whereas those of other mucosal tissues are wavelengths of around 410, 550, and 575 nm unlike the absorption spectrum shape of the gastric mucosa. Therefore, the excitation light with the wavelength of 505 nm may be effective for deep intramucosal cancer in other mucosal tissues because the absorption peak of mucosal tissues almost coincides with that of PpIX except for the wavelength of 505 nm.

## 5 Conclusion

The possibility of expanding the diagnostic depth for intramucosal cancer was investigated with a wavelength that excites the PpIX, which is distributed deeply more effectively than the wavelength of 405 nm used in the current ALA-PDD. The excitation wavelength that maximized the PpIX fluorescence intensity was derived at a deep fluorophore depth based on the numerical calculation, its effectiveness was evaluated in the *ex vivo* experiment. As a result, fluorescence of PpIX was detected with the wavelength of 505 nm at a deeper fluorophore depth than with the wavelength of 405 nm. These results suggest that the diagnosis depth of PDD with PpIX for intramucosal cancer can be extended by the 505-nm excitation light. As a future prospect, ALA-PDD using the excitation wavelength of 505 nm may identify intramucosal gastric cancer containing undifferentiated adenocarcinoma cells within a fibrous tissue. It is also expected that the excitation wavelength of 505 nm can be applied to the other mucosal tissues.
